# Supplementary figures and images for: Fluctuations in chromatin state at regulatory loci occur spontaneously under relaxed selection and are associated with epigenetically inherited variation in C. elegans gene expression
Source: PLoS Genet. 2023 Mar 2;19(3):e1010647. doi: 10.1371/journal.pgen.1010647 (PMC10013927; doi:10.1371/journal.pgen.1010647)

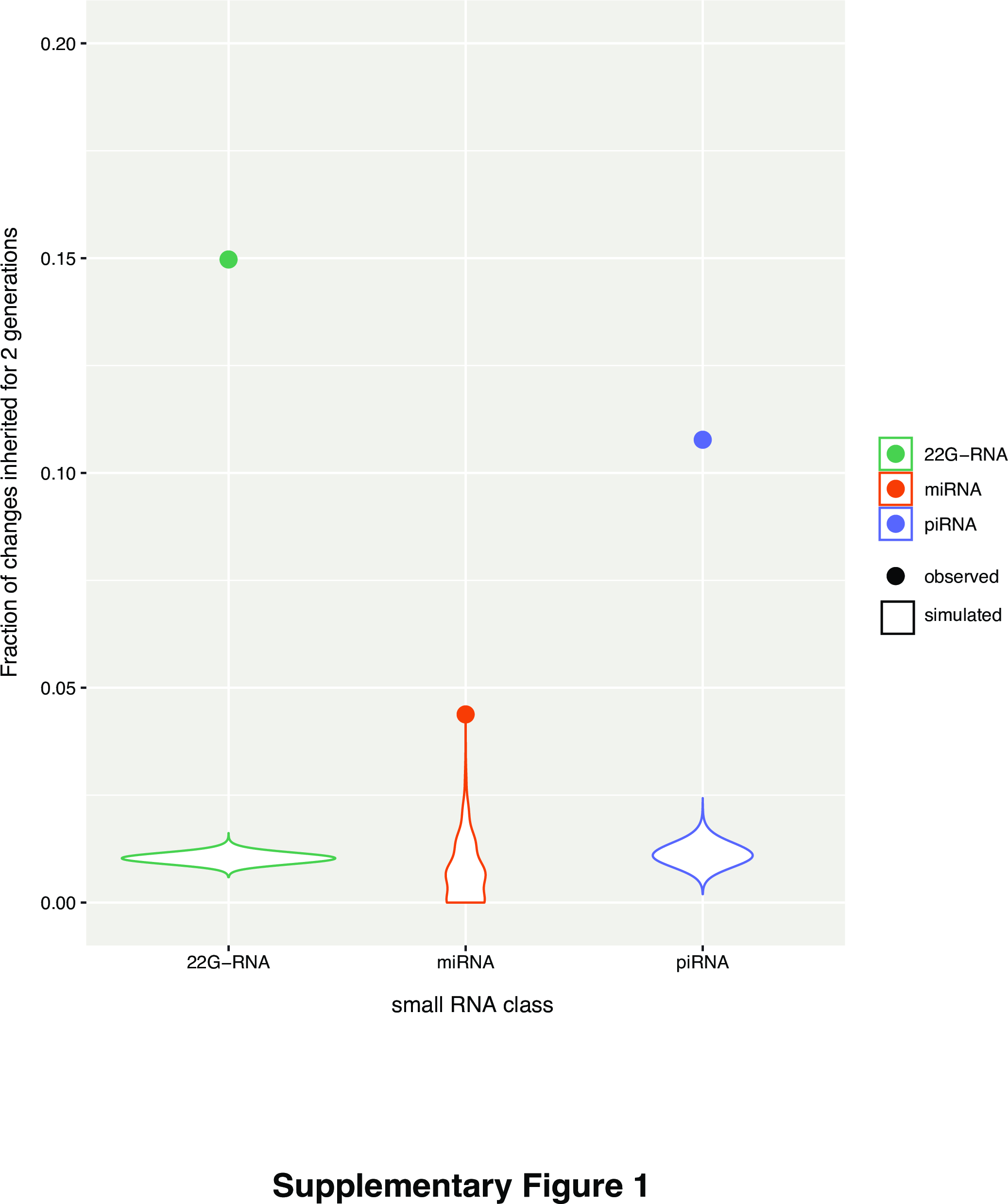

Supplement: S1 Fig — For each small RNA class (22G-RNAs, mi-RNAs and piRNAS), comparison of proportion of changes exceeding Z-score threshold (2.25, - 2.25) that are inherited to at least two subsequent generations between observed data (dot) and observations derived from 10000 random simulations (Methods). (TIF) [file pgen.1010647.s001.tif]

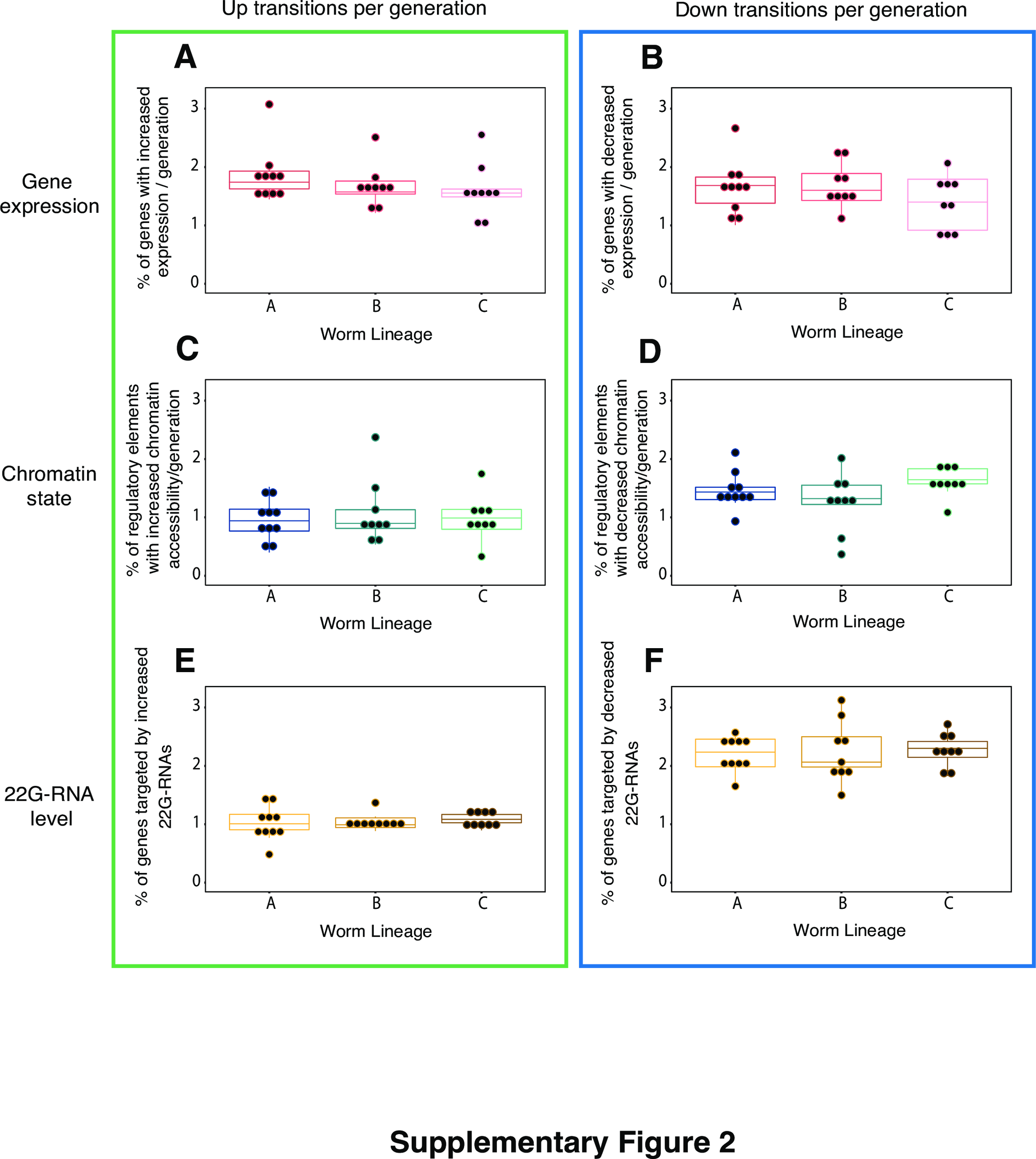

Supplement: S2 Fig — A & B. Gene expression changes. Percentage of genes showing significantly increased (A) and decreased (B) expression per generation. C & D. Chromatin state changes. Percentage of regulatory elements showing significantly increased (C) and decreased (D) chromatin accessibility per generation. E & F. 22G-RNA level changes. Percentage of genes in each lineage showing significantly increased (E) and decreased (F) antisense 22G-RNA levels per generation. For all plots, box shows the interquartile range with horizontal line at the median; the whiskers extend to the furthest point no more than 1.5 times the interquartile range. Kruskal-Wallis rank sum test gives non-significant p-values > 0.1 for comparison of worm lineages A, B and C within the categories of data type (Gene expression, Chromatin state, 22G-RNA level) and epimutation direction (Up transitions or Down transitions). (TIF) [file pgen.1010647.s002.tif]

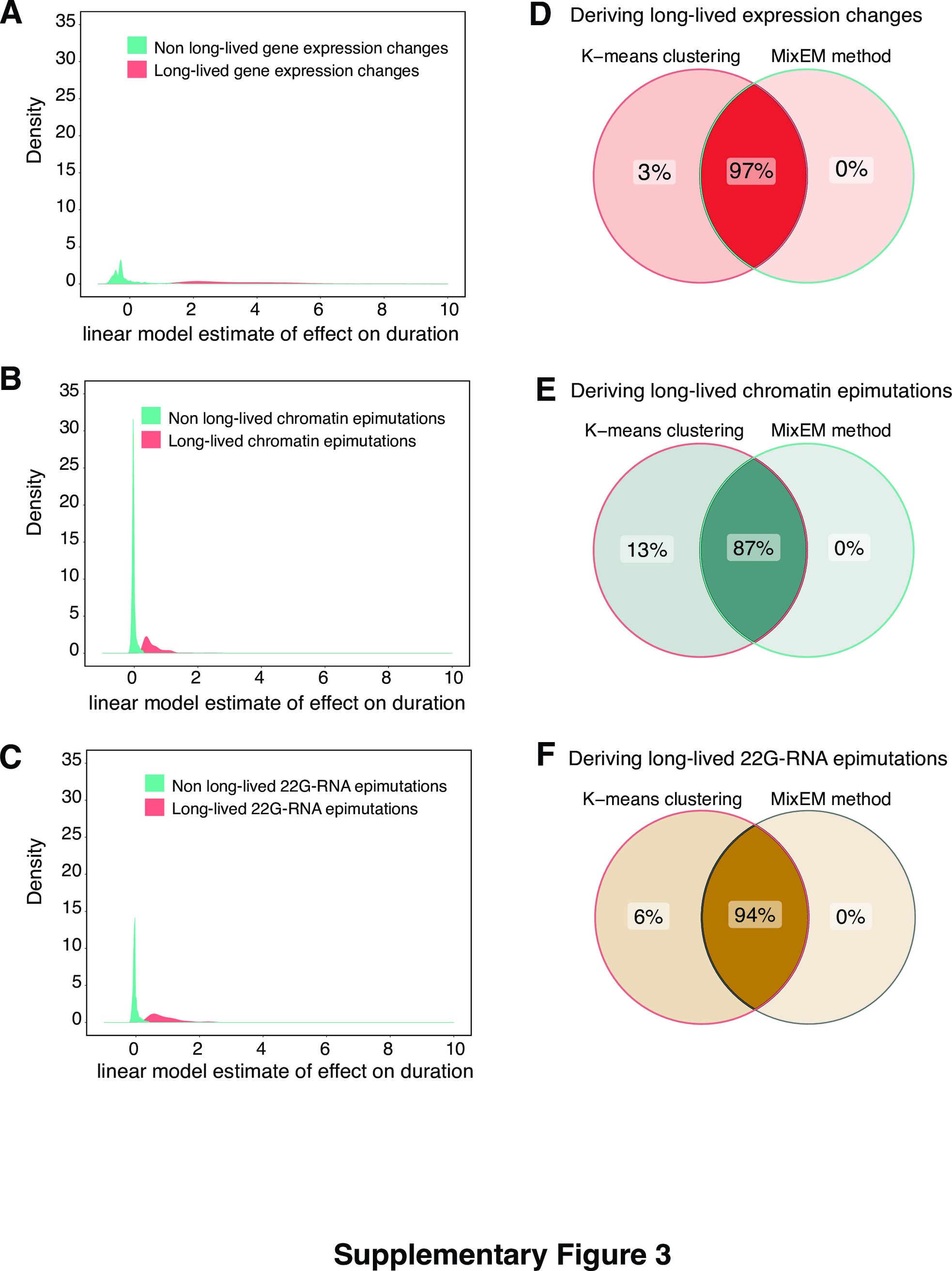

Supplement: S3 Fig — K-means clustering was done to identify the subset of genes predicted from a linear model to have significantly long-lived expression changes (A), chromatin-based epimutations (B), and 22G-RNA-based epimutations (C). The long-lived gene sets were derived independently using an alternative method based on expectation maximisation (EM) clustering using MixTools package in R (‘MixEM method’). The overlap between long-lived gene sets derived through both methods is shown for genes with expression changes (D), loci with chromatin-based epimutations (E), and loci with 22G-RNA-based epimutations (F). (TIF) [file pgen.1010647.s003.tif]

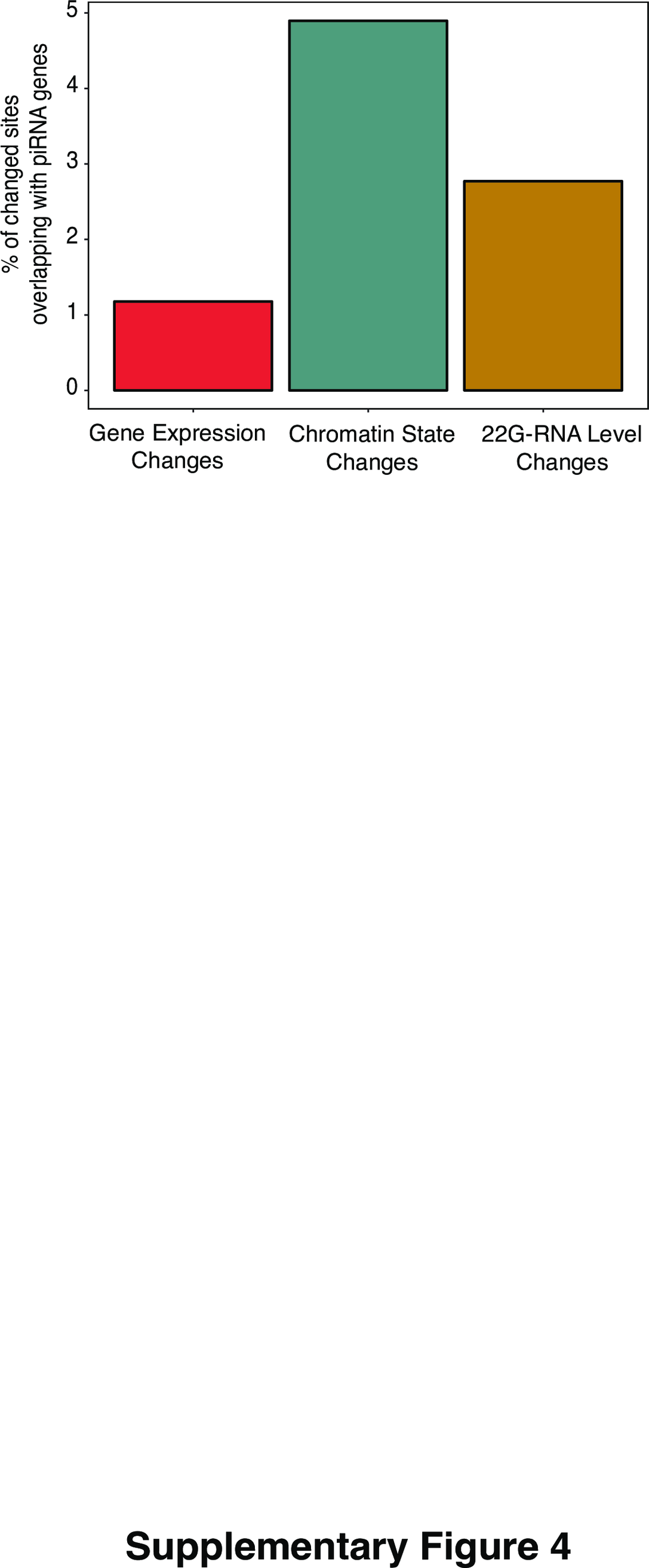

Supplement: S4 Fig — From left to right. Percentage of genes with expression changes (red bar), percentage of regulatory loci with chromatin epimutations (green bar) and percentage of genes with antisense 22G-RNA epimutations (gold bar) which overlap with piRNA cluster genes. (TIF) [file pgen.1010647.s004.tif]

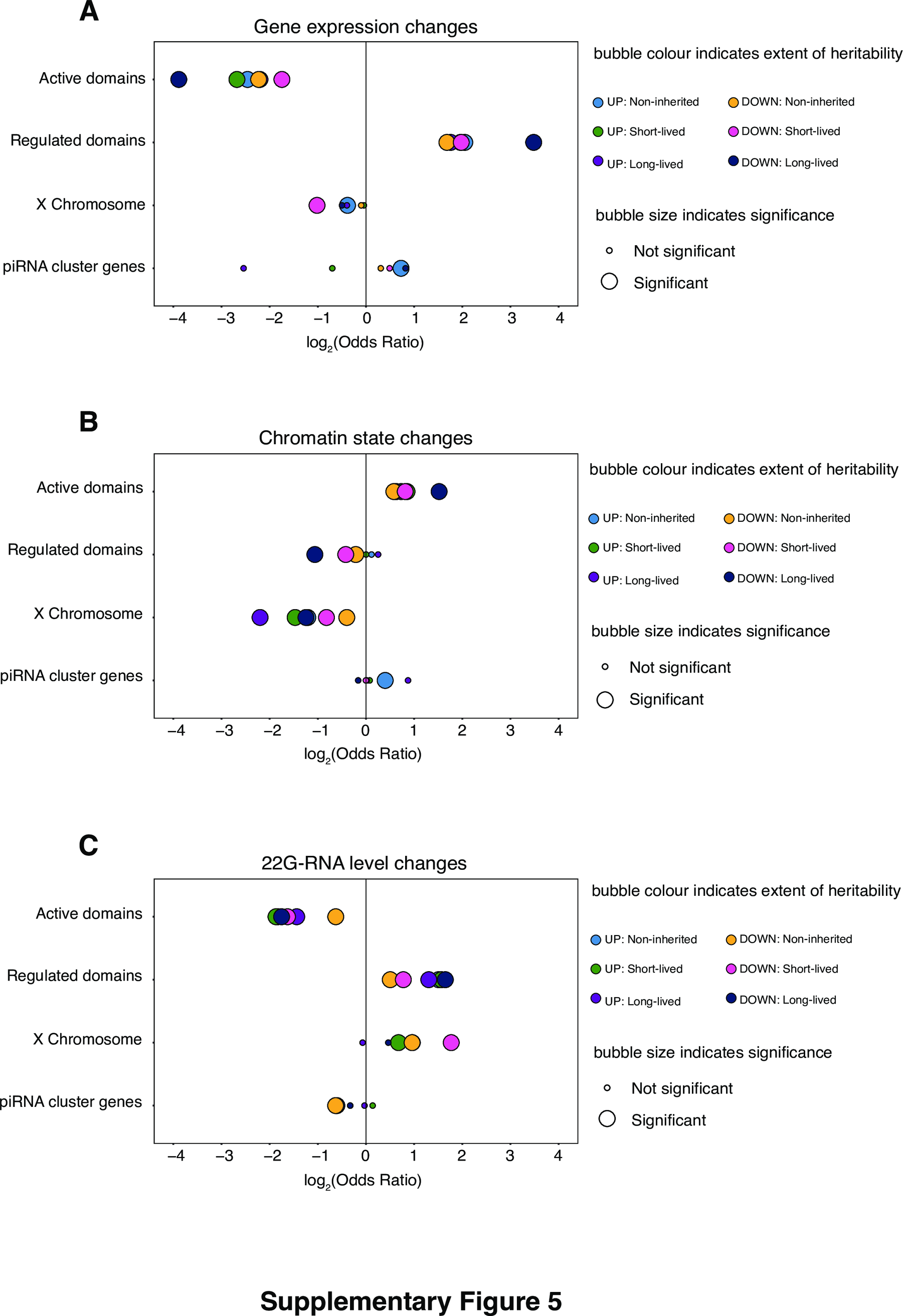

Supplement: S5 Fig — A. Bubble plot showing distribution of UP and DOWN non-inherited short-lived and long-lived gene expression changes in distinct chromatin domains. B. Bubble plot showing distribution of UP and DOWN non-inherited short-lived and long-lived chromatin-based epimutations in distinct chromatin domains. C. Bubble plot showing distribution of UP and DOWN non-inherited short-lived and long-lived 22G-RNA-based epimutations in distinct chromatin domains. For all plots, Y-axis shows constitutive chromatin domains investigated. X-axis shows log2(Odds) of enrichment. Odds ratios and p-values calculated with Fisher’s Exact Test with Bonferroni correction. p-value cut off for significance is 0.1. (TIF) [file pgen.1010647.s005.tif]

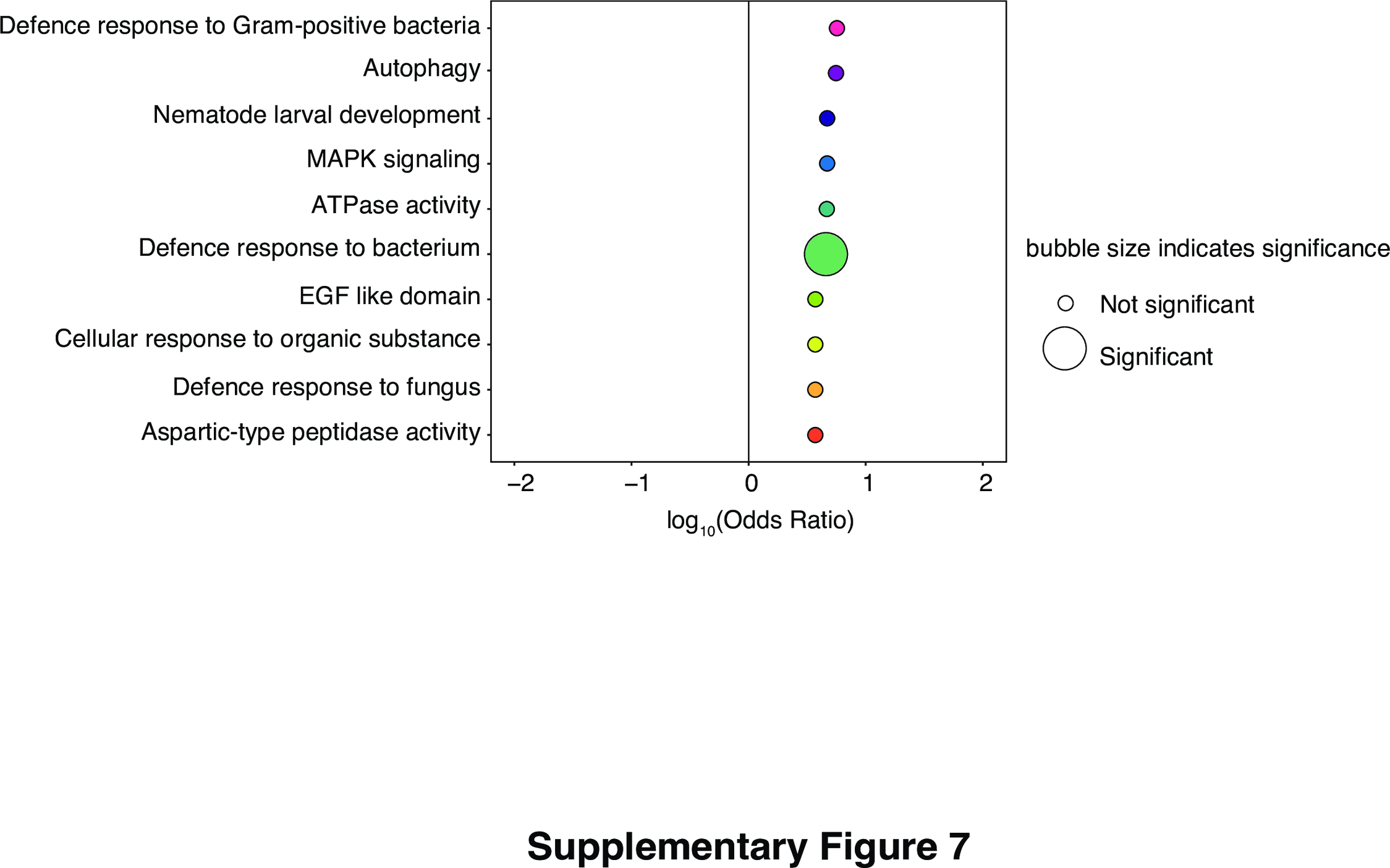

Supplement: S7 Fig — Y-axis shows ontology terms. X-axis shows log10(Odds) of enrichment. Top 10 results shown. Odds ratios and p-values are calculated using Fisher’s Exact Test with Bonferroni Correction. p-value cut off for significance is 0.1. (TIF) [file pgen.1010647.s007.tif]

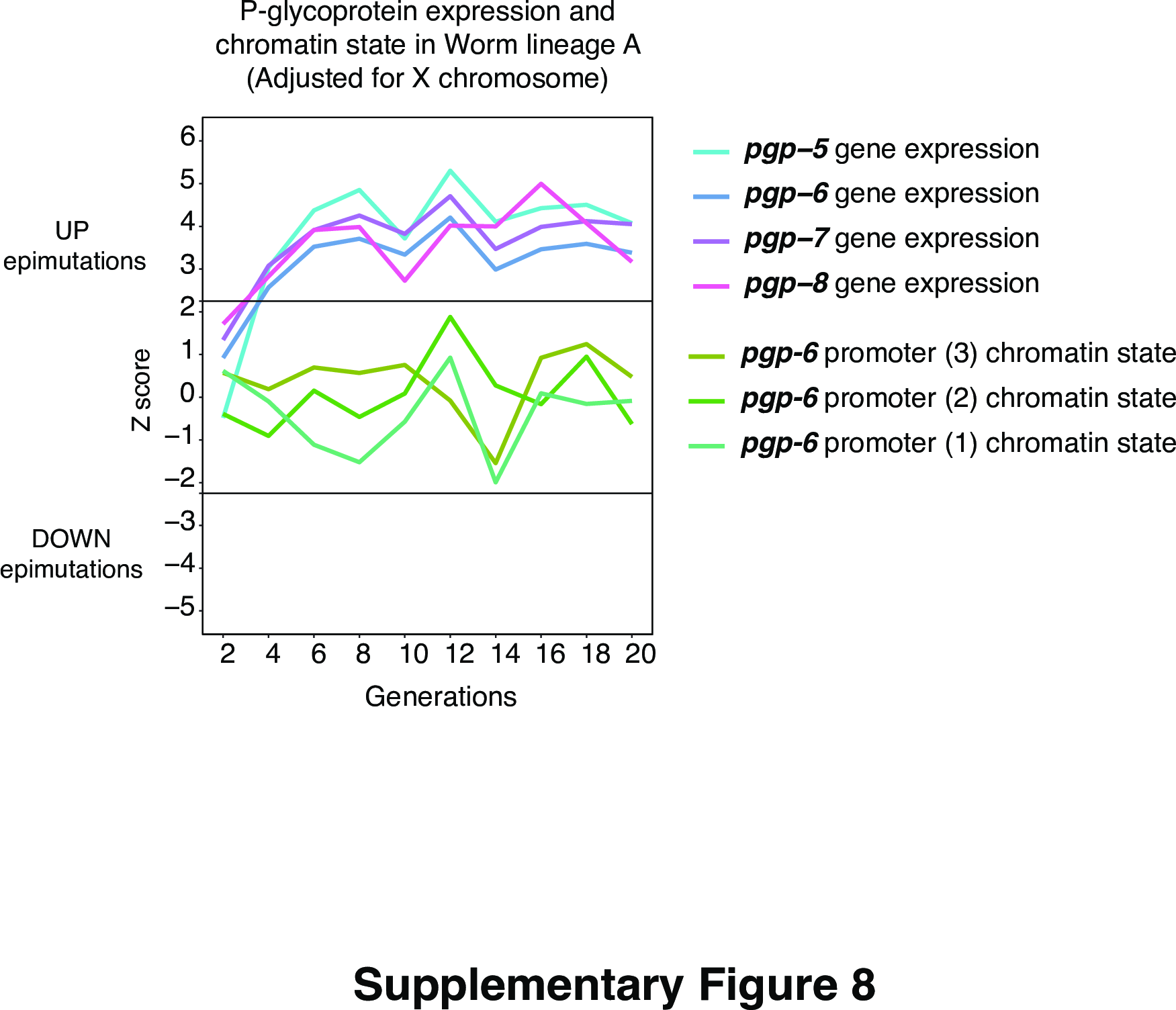

Supplement: S8 Fig — pgp-5, pgp-6, pgp-7, pgp-8 expression and regulatory element chromatin state over 20 generations. Normalisation of ATAC-seq counts is restricted to X chromosome. Data are for worm lineage A. Generational time points on X-axis. Z-score for epimutation status shown on Y-axis with 0 equivalent to PMA state. Horizontal thresholds indicate Z-score cut-offs; > 2.25 = UP epimutation and < - 2.25 = DOWN epimutation. (TIF) [file pgen.1010647.s008.tif]

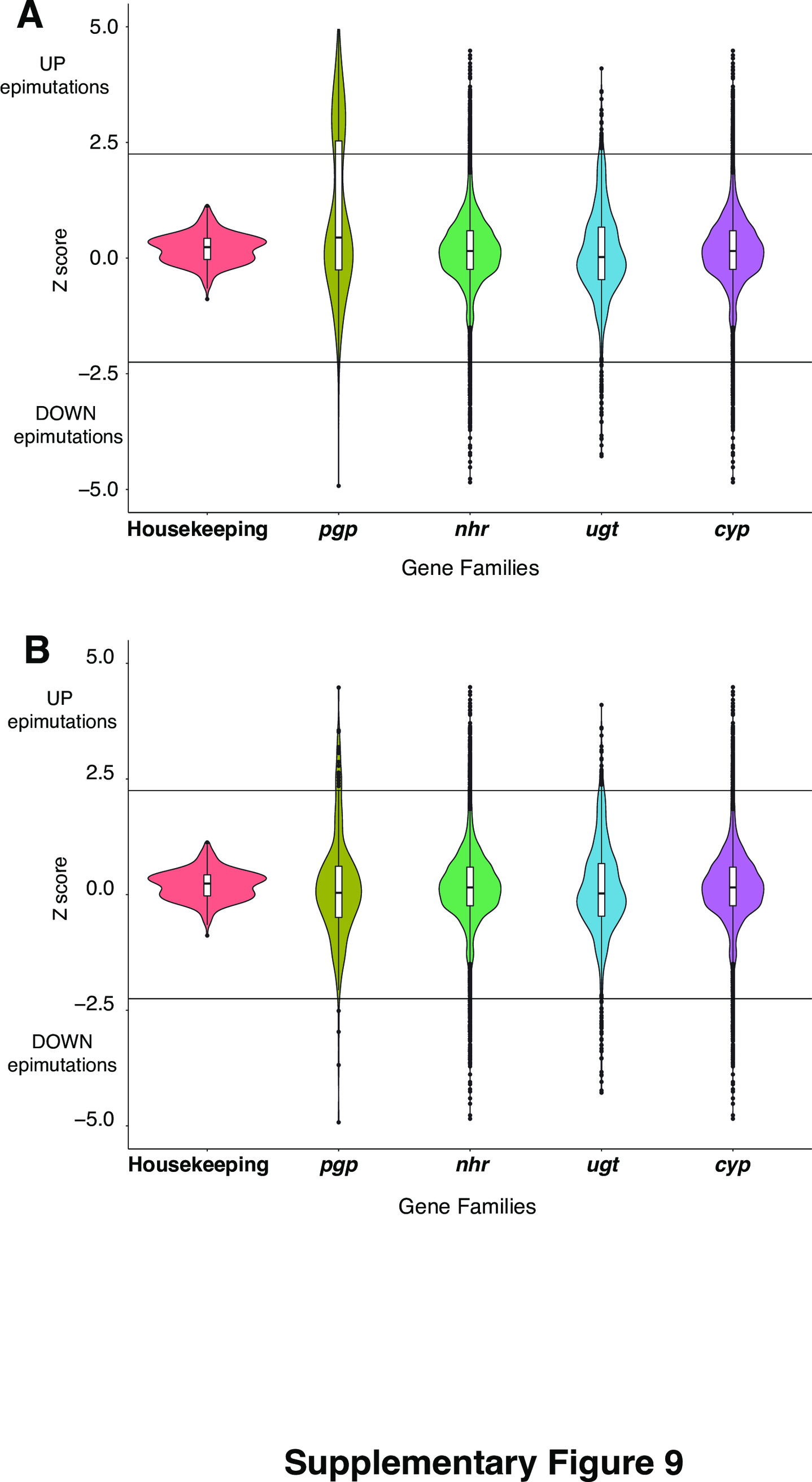

Supplement: S9 Fig — A. Expression over 20 generations across 3 independent worm lineages in housekeeping genes was compared to that of families of xenobiotic defence genes identified to contain long-lived epimutations. Housekeeping genes n = 13, pgp genes n = 14, nhr genes n = 268, ugt genes n = 66, cyp genes n = 74. B. As in (A) but the 4 gene pgp cluster (pgp-5, pgp-6, pgp-7, pgp-8) is removed showing that removing this gene cluster removes bias of pgp gene family towards Up epimutations as seen in (A). (TIF) [file pgen.1010647.s009.tif]
