# Supplementary material for: Fluctuations in chromatin state at regulatory loci occur spontaneously under relaxed selection and are associated with epigenetically inherited variation in C. elegans gene expression
Source: PLoS Genet. 2023 Mar 2;19(3):e1010647. doi: 10.1371/journal.pgen.1010647 (PMC10013927; doi:10.1371/journal.pgen.1010647)
Supplement: S6 Fig — Bubble plot showing enrichment of different small RNA pathways for 22G-RNA-based epimutations of different durations. Y-axis shows specific small-RNA pathway associated proteins. X-axis shows log2(Odds) of enrichment. Odds ratios and p-values are calculated using Fisher’s Exact Test with Bonferroni Correction. p-value cut off for significance is 0.1. (PDF) [file pgen.1010647.s006.pdf]

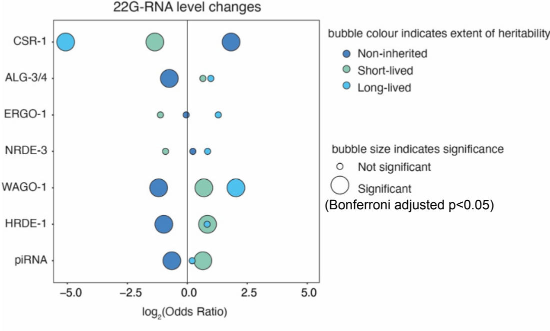
22G-RNA level changes
CSR-1
ALG-3/4
ERGO-1
NRDE-3
WAGO-1
HRDE-1
piRNA
−5.0
−2.5
0.0
2.5
5.0
log2(Odds Ratio)
bubble colour indicates extent of heritability
Non-inherited
Short-lived
Long-lived
bubble size indicates significance
Not significant
Significant
(Bonferroni adjusted p<0.05)
