# Supplementary material for: Fluctuations in chromatin state at regulatory loci occur spontaneously under relaxed selection and are associated with epigenetically inherited variation in C. elegans gene expression
Source: PLoS Genet. 2023 Mar 2;19(3):e1010647. doi: 10.1371/journal.pgen.1010647 (PMC10013927; doi:10.1371/journal.pgen.1010647)
Supplement: Attachment — Submitted filename: Responses_16_12_22_RW_PS.docx [file pgen.1010647.s067.docx]

**Point by point response to reviewers on Wilson *et al.* 2022**

**Reviewer #1:**

Minor:
Line 630: Update the paper to Johnson, L.M., Smith, O.J., Hahn, D.A., and Baer, C.F. (2020). Short-term heritable variation overwhelms 200 generations of mutational variance for metabolic traits in Caenorhabditis elegans. Evolution 74, 2451-2464. https://doi.org/10.1111/evo.14104.

**Authors:** *Thank you for pointing this out. We have updated accordingly (line 831-834)*

Figure 5: the two greens are too similar. Use colours that are easier to distinguish.

**Authors:** *We have now changed the colour for ‘long-lived’ on both figures 4 and 5 to make the 3 categories easier to distinguish in both figures*

**Reviewer #2:**

Points for improvement:

1) In Figure 4 it will be informative to see the directionality of changes: whether “UP” or “DOWN” changes drive the enrichment or depletion.

**Authors:** *We thank the reviewer for this suggestion.  We have now included an analysis where we examined epimutations separately for both directions.  In all cases the trends were similar to when both directions were considered together.  We have included this data as a new supplemental figure (Supp. Fig X) and an additional sentence in the manuscript.*

*The relevant section in the corresponding main text (lines 396 – 398) now reads:*

Stratification of epimutations by whether they were UP or DOWN did not affect the overall trends, suggesting that both directions contribute to the enrichments observed for all types of epimutation analysed (Supp. Fig. 5).

2) Figure 1C legend or corresponding text. It should be spelled out that “UP” counts of ATAC-seq correspond to increased opening of chromatin and “DOWN” counts reflect chromatin closing.

**Authors:** *We have included additional information in both Figure 1 legend and in the corresponding main text to improve the clarity of this.*

*The relevant section in the Figure 1 legend (lines 127 -130) now reads:*

**C.** Identification of chromatin-based epimutations. Epimutated loci were defined as regulatory element loci with ATAC-seq counts (rings on plot) that were significantly greater (UP) or lower (DOWN) than that of the same locus in the PMA generation according to a Z-score cut off. UP epimutations correspond to increased opening of chromatin while DOWN epimutations reflect chromatin closing.

*The relevant section in the corresponding main text (lines 171 – 175) now reads:*

Therefore, increased ATAC-seq signals (Z > 2.25) were ‘UP’ chromatin-based epimutations, indicating more open chromatin states. Decreased ATAC-seq signals (Z < -2.25) were ‘DOWN’ chromatin-based epimutations, indicating more closed chromatin states. We took the same approach to identify small RNA-based epimutations, for which ‘UP’ indicated increased small RNA levels while ‘DOWN’ indicated decreased small RNA levels.

3) There should be more precision in describing what is subject to epimutation (22G-RNA abundance, gene expression, chromatin accessibility) without implying functional consequences. This is most relevant to 22G-RNAs. Some examples are below.

**Authors:** *We have further clarified this by explaining that small RNA-based epimutations constitute significantly increased or decreased small RNA levels and chromatin-based epimutations constitute significantly more open or more closed chromatin states.*

*The relevant section of text (lines 171 – 175) now reads (as for response to point 2):*

Therefore, increased ATAC-seq signals (Z > 2.25) were ‘UP’ chromatin-based epimutations, indicating more open chromatin states. Decreased ATAC-seq signals (Z < -2.25) were ‘DOWN’ chromatin-based epimutations, indicating more closed chromatin states. We took the same approach to identify small RNA-based epimutations, for which ‘UP’ indicated increased small RNA levels while ‘DOWN’ indicated decreased small RNA levels

Line 12: “Changes in epigenetic regulation termed epimutations”. I suggest: ““Changes in epigenetic regulators…”

**Authors:** *We have made this revision as suggested.*

Line 14: “Epimutations mediated by small non-coding RNAs”. What are those epimutations: gene expression changes? Phenotypes? Corrections: “Epimutations in 22G-RNA pools” or “epimutations carried by small non-coding RNAs”.

**Authors:** *We agree, the use of the word ‘mediated’ was potentially confusing. We have revised the text to remove this and instead have used the following expressions throughout the text of the manuscript: small RNA-based epimutations, chromatin-based epimutations and 22G-RNA-based epimutations. (See throughout text).*

Also on line 67: “epimutations mediated by 22G-RNAs arise spontaneously”. Rather “epimutations in 22G-RNA levels”, “epimutations driving 22G-RNA abundance arise spontaneously”.

**Authors:** *We have revised this line as suggested.*

*The relevant section of text (line 66-67) now reads:*

We demonstrated that 22G-RNA-based epimutations arise spontaneously in *C. elegans* MA lines (Beltran et al., 2020)*.*

Line 505: “… 22G-RNAs drive spontaneous short-lived epimutations” is not correct based on the cited publication. Instead: “22G-RNA levels are subject to spontaneous short-lived epimutations”.

**Authors:** *We have revised this line as suggested.*

*The relevant section of text (lines 550 – 554) now reads:*

This expands our previous work demonstrating that 22G-RNA levels are subject to spontaneous short-lived epimutations in *C. elegans* (Beltran et al., 2020) It also fits with recent results showing that short-term heritable variation in metabolite levels occurs in C. elegans lines propagated with limited population size (Baer et al., 2020).

Lines 134, 136, 221, Fig 2 legend: “chromatin mediated epimutations”. Chromatin mediates epimutations of what? Correct: “epimutations in chromatin accessibility” or “epimutations carried via chromatin organization”.

**Authors:** *As above we have now changed ‘chromatin mediated epimutations’ throughout the text to ‘chromatin-based epimutations’*

Line 17: “mechanism for transgenerational epigenetic inheritance.” Inheritance of what? Apparently, gene expression changes, based on the manuscript. Traditionally, epigenetic inheritance of phenotypes is implied.

**Authors:** *We have revised this line as suggested. This section (lines 15-17) now reads:*

Here, we explored whether chromatin states also undergo spontaneous change and whether this could be a potential alternative mechanism for transgenerational inheritance of gene expression changes.

Line 226: “epimutations in piRNA loci” change to “epimutations in piRNA expression levels”

**Authors:** *We have revised this line as suggested.*

*The relevant section of text (lines 229 – 232) now reads:*

Heritable epimutations in miRNA expression levels were very rare, although interestingly slightly above what would be expected by chance. Heritable epimutations in piRNA expression levels were more common but affected fewer loci than changes in 22G-RNAs mapping to protein-coding genes (Supp. Fig. 1).

4) Discussion relevant to Figure 7 can be more organized. Line 599-600 and Figure 7: “mechanism of epimutation inheritance itself may become more robust under stress” is vague, suggest how this may happen. For example, Scenario 1 is consistent with induced epimutations causing specific changes in gene expression that become more frequent in the population and more heritable. Scenario 2 reflects selection of existing advantageous epimutations.

**Authors:** *We have revised this section for better organisation and clarity as advised. In addition, we have included the suggestion that epimutation inheritance may become more robust under stress due to enhanced self-templating of epimutations and referenced Zhang 2015.*

*The relevant section of text (lines 641 – 647) now reads:*

Under conditions of xenobiotic stress, the propagation of pre-existing epimutations which are now advantageous in this setting is enhanced due to selection. On removal of the stressor, epimutations no longer provide a survival advantage, or indeed may be detrimental. Selection then ceases to drive propagation of the epimutation (Fig. 7). Alternatively, the mechanism of epimutation inheritance itself may become more robust under stress, for example due to enhanced ‘self-templating’ of the epimutation (T. Zhang et al., 2015), leading to population expansion of an epimutation which may or may not have adaptive benefit.

5) I suggest that the authors provide data tables in a more accessible format, such as Excel.

**Authors:** *We have now produced .csv files which can be viewed in Excel but suggest that this is in addition to the .RData objects we have also supplied as these can be directly used with the R code we provided.*

**Reviewer #3:**

Experimental and Major Comments:

1. Figure 1C introduces the criteria and thresholds of the ATAC-seq data by which changes in chromatin are considered a “chromatin epimutation” for subsequent analysis. This figure also introduces UP and DOWN as categories of chromatin state epimutation, but the text does not place these in functional context. What do these designations mean for how chromatin accessibility has changed? Are we to assume that an UP epimutation indicates more accessible chromatin and DOWN epimutation indicates more closed? The functional conclusions of 1C are not thoroughly discussed, even though they are critical for interpreting the subsequent data.

**Authors:** *We have now addressed this by clarifying the functional meaning of chromatin and small RNA based epimutations as increases and decreases in chromatin accessibility or small RNA level; please see also response to Reviewer 2.*

*This section in Figure 1 legend (lines 127 -130) now reads:*

**C.** Identification of chromatin-based epimutations. Epimutated loci were defined as regulatory element loci with ATAC-seq counts (rings on plot) that were significantly greater (UP) or lower (DOWN) than that of the same locus in the PMA generation according to a Z-score cut off. UP epimutations correspond to increased opening of chromatin while DOWN epimutations reflect chromatin closing.

*The relevant section in the corresponding main text (lines 171 – 175) now reads:*

Therefore, increased ATAC-seq signals (Z > 2.25) were ‘UP’ chromatin-based epimutations, indicating more open chromatin states. Decreased ATAC-seq signals (Z < -2.25) were ‘DOWN’ chromatin-based epimutations, indicating more closed chromatin states. We took the same approach to identify small RNA-based epimutations, for which ‘UP’ indicated increased small RNA levels while ‘DOWN’ indicated decreased small RNA levels.

a. Especially regarding Figure 3 and Table 1, the introduction of discordant vs concordant UP and DOWN chromatin changes is extremely confusing, in part due to incomplete explanation. Table 1 as a stepwise analysis of gene expression vs chromatin state is not well explained – the “Background” column is not defined in-text, nor is it clear what the “Background” categories mean relative to the “Association tested” category, nor is it adequately discussed how to interpret these results. The calculations performed to get the reported “Odds ratio” is not well defined. While the basic conclusions drawn in Figure 3B do appear clear and robust, Table 1 requires that we simply “take the author’s word for it” because, as currently presented, it is nearly impossible to interpret.

b. The above criticism holds for Table 2 and Figure 3C, which performs a similar analysis comparing gene expression changes to 22G-RNA epimutations.

**Authors:** *We thank the reviewer for these helpful comments. We have now more clearly referenced the background gene set which changes in a stepwise manner as our analysis progresses and explaining simply how the calculation is performed at each stage, while the table legends indicate that formally this was done using Fishers Exact Test. We have also included an additional ‘Test’ number column in both tables to more clearly refer the reader to each analysis in turn as we discuss the results. We have now included additional discussion and interpretation.*

*Regarding the main text description of Fig 3 and Table 1, the relevant text (lines 310- 321) now reads:*

To test this, we conducted a stepwise analysis of the association between chromatin-based epimutations and expression changes. We first looked at a background of all genes with annotated regulatory elements regardless of whether they had expression changes or not. The proportion of the genes which had expression changes was compared to the proportion of the genes which had chromatin-based epimutations. The overlap, in which genes had both expression changes and epimutations, was calculated. At this level, we found that chromatin-based epimutations rarely accompanied gene expression changes (Tests 1 & 2, Table 1).

We then looked more closely within the subset of genes which had expression changes. In this background we found a strong association between inherited expression changes and simultaneous chromatin-based epimutations (Fig. 3B, Tests 3 & 4, Table 1).

*Regarding the main text description of Fig 3 and Table 2, the relevant text (lines 329 -333) now reads:*

Using the equivalent stepwise approach for 22G-RNAs, we found that changes in 22G-RNAs mapping antisense to protein-coding genes overlapped more strongly with changes in gene expression in a background of all genes (Test 1, Table 2), and as with chromatin, genes showing heritable changes in gene expression were significantly enriched for simultaneous 22G-RNA-based epimutations (Fig. 3C, Tests 3 & 4, Table 2)

*Regarding additional interpretation and discussion on these findings, the relevant text (lines 340-344) now reads:*

Therefore, although chromatin-based epimutations may occur without concurrent expression changes, on occasions where heritable expression changes are observed, simultaneous chromatin-based epimutations tend to accompany these. Chromatin-based epimutations may produce a favourable environment for changes in gene expression to be inherited but additional factors such as 22G-RNAs may be required to ‘license’ these.

*Regarding the definition of the terms ‘concordant’ and ‘discordant’, we have revised the text for better clarity and have explicitly stated what these terms mean for both chromatin and small RNAs*

*For chromatin, the relevant text (lines 346 – 349) now reads:*

We examined whether chromatin-based epimutations occurring in the same generation (simultaneous) and in the same direction (UP/UP or DOWN/DOWN) as gene expression changes i.e. ‘concordant’ had any effect on the odds that expression changes would be inherited, compared to simultaneous but ‘discordant’ (UP/DOWN, DOWN/UP) changes.

*For small RNAs, the relevant text (lines 353 – 355) now reads:*

In the case of genes with simultaneous 22G-RNA-based epimutations and gene expression changes, we found that both concordant (UP/UP or DOWN/DOWN) and discordant (UP/DOWN, DOWN/UP) 22G-RNA-based epimutations were similarly enriched for inherited expression changes (Fig. 3E).

c. Figures 3D and 3E both build from the observations in Tables 1 and 2. Now the authors present pairwise analysis between UP and DOWN trends (presumably with reference to Fig 2C, although 2C only discusses chromatin changes and here they also include gene expression changes and 22G-RNA changes), and “concordant” vs “discordant” changes (in reference to whether the compared changes occur in the same direction) to observe the odds of any 2 simultaneous epimutation events being inherited. Contextualizing what UP and DOWN functionally mean for a concordant or discordant change in 1) gene expression, 2) chromatin, and 3) 22G-RNA epimutations is essential to interpreting this figure and to supporting the conclusions of the paper.

**Authors:** *We have improved clarity on this issue by explaining more clearly that ‘concordance’ means a simultaneous (occurring in same generation) epimutation for which the change, whether in chromatin state or small RNA level is in the same direction i.e. UP/UP or DOWN/DOWN as the gene expression change, while for discordant epimutations, the direction of change between the epimutation and the gene expression is mismatched (UP/DOWN or DOWN/UP).*

*For chromatin, the relevant text (lines 346 – 349) now reads:*

We examined whether chromatin-based epimutations occurring in the same generation (simultaneous) and in the same direction (UP/UP or DOWN/DOWN) as gene expression changes i.e. ‘concordant’ had any effect on the odds that expression changes would be inherited, compared to simultaneous but ‘discordant’ (UP/DOWN, DOWN/UP) changes.

*For small RNAs, the relevant text (lines 353 – 356) now reads:*

In the case of genes with simultaneous 22G-RNA-based epimutations and gene expression changes, we found that both concordant (UP/UP or DOWN/DOWN) and discordant (UP/DOWN, DOWN/UP) 22G-RNA-based epimutations were similarly enriched for inherited expression changes (Fig. 3E).

2. Lines 387-391 indicate that epimutations at 22G-RNA level (Fig 4C) at the X chromosome are enriched. According to the figure, “non-inherited” and “short lived” are enriched, but “long-lived” are close to zero and not significant. Lines 390-391 state “Both long-lived and short-lived epimutations were similarly enriched” and it is unclear if this still refers specifically to the 22G-RNA level changes at the X chromosome (per the previous sentence), but if so, then it is untrue.

**Authors:** *We have corrected and updated this line to: ‘enriched for both non-inherited and short-lived 22G-RNA-based epimutations’. We have referred again to Fig 4C.*

*The relevant text (lines 385 – 388) now reads:*

The X chromosome, which is subject to H4K20 methylation as a result of dosage compensation and does not show high levels of either H3K27me3 or H3K36me3 (Lau et al., 2014; Lau & Csankovszki, 2015; Meyer, 2022), was also enriched for both non-inherited and short-lived 22G-RNA-based epimutations (Fig. 4C).

3. The paragraph starting on line 393 discusses enrichment trends in the chromatin state change epimutations (Figure 4B). It states “piRNA clusters… were strongly enriched for epimutations at the level of chromatin.” – it’s unclear if this refers to directly to Figure 4B, but if so it does not appear supported by the data. The only bubble visible for piRNA cluster genes under chromatin state changes (bottom of 4B) is for “non-inherited” and it lies very close to zero, indicating it is not a strongly enriched category for any inherited changes at all. Further,

4. This paragraph also refers to Supplemental Figure 4 which does in fact appear to support their stated conclusion, although this suggests a conclusion contrary to Figure 4B, so acknowledgement of where that discrepancy arises would be important.

**Authors:** *Re points 3 & 4. We have re-made these plots (Fig 4) and have changed the range of the X axis from -5 🡪 5 to -3 🡪 3 which gives better resolution and therefore the enrichment of chromatin-based epimutations in piRNA cluster genes (Fig 4 B) is more easily discernible. We have also clarified the relevant text.*

*The relevant text (lines 390- 396) now reads:*

Interestingly, we noticed that piRNA clusters, which occupy two broad regions on chromosome IV (Batista et al., 2008; Das et al., 2008; Ruby et al., 2006) were enriched for non-inherited chromatin-based epimutations (Fig. 4B). This was surprising because piRNAs are predominantly produced from H3K27me3-marked regions (Beltran et al., 2019). Indeed, the proportion of chromatin epimutations in the piRNA clusters compared to the wider genome was greater than that of 22G-RNA-based epimutations and gene expression changes (Supp. Fig. 4).

5. Figure 5 identifies the cellular pathways enriched for epimutations associated with gene expression changes (5A), chromatin state changes (5B), and 22G-RNA level changes (5C), and sets up for the conclusion that environmental stress and pathogen responses could be an evolutionary reason to allow for high epimutation rates under normal growth conditions. However, these defense pathways are only 1 among many different pathways with equivalent enrichment, yet the others are not particularly discussed. I think the fact that across an array of pathways, “long-lived” epimutations are very highly enriched as compared to “non-inherited” or “short-lived” specifically at the chromatin state level (5B) is extremely interesting and perhaps should be emphasized. This strongly supports how chromatin-based epigenetic changes can set the stage for heritable changes lasting over 10-generations (compare this to 5A, for example, where “short-lived” and “long-lived” changes behave similarly for gene expression changes).

**Authors**: *We thank the reviewer for this encouraging interpretation of our results. We have expanded this section as per Reviewer suggestion to reflect the enrichment of long-lived chromatin-based epimutations across an array of pathways and have highlighted the enrichment in SET domains as interesting in addition to defence response to bacteria and have referenced Herbette 2017. We have included the idea that chromatin-based epimutations establish conducive environments for heritable expression changes to occur which may have wide ranging phenotype effects.*

*The relevant text (lines 432 – 440) now reads:*

Compared to non-inherited and short-lived categories, long-lived chromatin-based epimutations at regulatory elements were strongly enriched across an array of pathways (Fig. 5B), including regulation of defence response to bacterium. This finding is surprising given the previous observation (Fig. 4A & 4B) that gene expression changes and chromatin epimutations are predominantly in distinct chromatin domains. Additionally, we were interested to see enrichment in germ-line stabilising SET domain pathways (Herbette et al., 2017), and other pathways involving translation, protein localization and development. This supports the notion that chromatin-based epimutations establish conducive environments for heritable expression changes to occur which may have wide ranging phenotype effects.

6. Figure 6 identifies a cluster of P-glycoprotein genes that previous studies have already shown to be expressed and behave similarly, and then shows that they behave similarly with expression patterns that change as a unit, as expected. Expanded upon below, Figure 6 is an interesting observation but lends little support to the full article’s conclusions, especially with regards to chromatin state epimutations.

a. The authors have no testable explanation as for why this cluster of genes suddenly increased in expression upon onset of the experiment in all 3 worm lineages (6C), which makes it an interesting observation but I’m unsure how it can be used to support their conclusions.

b. Despite this unexplained robust increase in expression, none of the simultaneously observed chromatin changes at the cluster pass the threshold for UP or DOWN (6C). I recognize that they address this in the text, stating that chromatin activity still “appeared to broadly track with the peaks and troughs of gene expression change” (line488-489), but that association appears weak and overall does not particularly support their conclusions that changes in chromatin state are associated with epimutations in gene expression level.

c. Further, they go on to try and address this discrepancy by adjusting their threshold settings, and this still does not appear to make even a single time point for promoter 1, 2, or 3 chromatin states pass the Z-score significance threshold (supplemental figure 6C).

d. In fact, Lines 484-485 state “We did not observe any epimutations in 22G-RNAs that could explain this”, referring to the co-regulation of this P-gp cluster. Yet, supplemental figure 6B shows a steep drop in 22G-RNAs targeting this cluster that corresponds exactly in time with the increase in cluster gene expression. This dip in 22G-RNAs also does not pass the Z-score threshold (as the chromatin state changes did not either), but appears it may account for the change in cluster expression more robustly than the weak correlation observed with the peaks and troughs in chromatin state.

**Authors:** *Re points 6 a, b, c & d.* *We agree with the Reviewer’s important points here. We have reframed this section and revised Figure 6. We now present a comparison between chromatin behaviour and 22G-RNA behaviour in relationship to pgp gene expression patterns. We have emphasized that chromatin changes are not strongly associated with changes in pgp expression. Rather we highlight that 22G level changes appear to be more in sync with expression changes than chromatin state changes and suggest that these may contribute to the changes in gene expression.*

*We have also revised the colours used in Figure 6A to make the trends in chromatin state at pgp promoters more distinguishable*

*The relevant section of text (lines 528- 545) now reads:*

Although not matching the threshold for epimutations, we observed that chromatin activity at the three regulatory elements for *pgp-*6 appeared to broadly track with some of the changes in gene expression for *pgp* genes 5, 6, 7, and 8 (Fig. 6A). We wondered whether the genome-wide threshold for qualifying a chromatin state change as an epimutation under-estimated the extent of changes in the setting of the X chromosome due to its unique chromatin environment (Meyer, 2022). To address this, we derived Z-scores for X chromosome gene expression and chromatin state change loci using the same method as before but restricting the distribution of data points to those only derived from the X chromosome in order to produce X chromosome-specific Z-scores. However, the effect of adjusting for the specific context of the X chromosome was minimal (Supp. Fig. 8). Interestingly, we observed that a steep decline in 22G-RNAs occurred at precisely at the same time as the initial steep increase in gene expression, whilst chromatin accessibility did not behave similarly (Fig. 6B). In subsequent generations however, the fluctuations in chromatin state changes appeared to correlate better with gene expression changes (Fig. 6A). It is possible therefore that chromatin and 22G-RNA levels can influence gene expression at this locus independently. However, it is important to note that neither the 22G-RNAs nor changes in chromatin are sufficient to completely explain the gene expression changes, suggesting that additional regulatory factors may be involved in regulating the heritable changes in gene expression that we observed at these loci.

Minor Comments:
1. The authors should address the RNAi pathways represented in their 22G-RNA epimutation data. Their previous publication (Beltran 2020) states that canonical silencing pathways (including piRNA, HRDE-1, WAGO-1, ERGO-1, and NRDE-3) were enriched for 22G-RNA epimutations but that the canonical licensing pathway CSR-1 is not. If this remains true, it should be briefly discussed here as well as it is important for interpreting the functional relevance of concordant and discordant changes in gene expression vs 22G-RNA epimutations.

**Authors:** *We agree that this is relevant and have now undertaken this additional analysis. Additionally, we include a further supplementary figure (Supp. 4B) to reflect our findings which indeed recapitulate those of our previous publication (Beltran et al. 2020). We have suggested that epimutations targeting both activating and silencing small RNA pathways is a possible explanation for why gene expression changes were similarly enriched for both concordant and discordant 22G-RNA-based epimutations (Fig 3E).*

*The relevant section of text (lines 400- 412) now reads:*

In previous work we investigated stability of 22G-RNA-based epimutations targeting genes associated with different small RNA pathways (Beltran et al., 2020). We showed that epimutations targeting the CSR-1 pathway, which is associated with gene activation (Seth et al., 2013), were unstable and short-lived, while those targeting the WAGO-1 pathway, which drives gene silencing (Gu et al., 2009), were highly stable, at times persisting beyond 10 generations. Having identified 22G-RNA-based epimutations with differing durations in this work, we then checked for differential associations with various small RNA pathways. We found the same enrichment pattern as in our previous work. Targets of non-inherited 22G-RNA-based epimutations strongly overlapped with CSR-1 pathway targets while targets of long-lived 22G-RNA-based epimutations strongly overlapped with WAGO-1 pathway targets (Supp. Fig. 6). Epimutations targeting both activating and silencing small RNA pathways is a possible explanation for why gene expression changes were similarly enriched for both concordant and discordant 22G-RNA-based epimutations (Fig 3E).

2. Figures 4 and 5: the bubbles indicating “non-enriched”, “short-lived”, and “long-lived” heritability are so close in color as to be difficult to tell apart. Additionally, the dots indicating “not significant” should still be visibly distinguishable and color coordinated.

**Authors:** *We have revised all relevant figures throughout the manuscript. The colour for ‘long-lived’ categories has been changed to light blue. Additionally, non-significant results which had been presented as transparent bubbles are now shown as solid colours. (see Figures: 3 D&E 4, 5, supp. Fig 4B, supp. Fig 5).*

3. Line 452-453 references a conclusion drawn from a “previous observation” – what figure does this refer to?

**Authors:** *This refers to Fig. 4A and 4B and we have now referenced this accordingly.*

*The relevant section of text (lines 432- 436) now reads:*

Compared to non-inherited and short-lived categories, long-lived chromatin-based epimutations at regulatory elements were strongly enriched across an array of pathways (Fig. 5B), including regulation of defence response to bacterium. This finding is surprising given the previous observation (Fig. 4A & 4B) that gene expression changes and chromatin epimutations are predominantly in distinct chromatin domains.

4. Supplemental Figure 6C is not included in the figure legend.

**Authors:** *We have now revised this figure as discussed in response to point 6. (see Revised Figure 6 and revised Supplementary Fig. 6)*
